# Campylobacter Pouchitis Mimicking the Appearance of Crohn's Disease

**DOI:** 10.1155/2016/5254914

**Published:** 2016-07-31

**Authors:** Greg S. Cohen, Meena Prasad

**Affiliations:** Department of Medicine, Division of Gastroenterology, Northwestern University, Chicago, IL 60611, USA

## Abstract

An unusual case of campylobacter pouchitis resembling the endoscopic appearance of Crohn's disease is reported.

## 1. Introduction

Proctocolectomy with ileal pouch-anal anastomosis (IPAA) is the preferred treatment for medically refractory ulcerative colitis (UC), UC-associated dysplasia or malignancy, and familial adenomatous polyposis. Postoperative complications of IPAA include pouchitis, Crohn's disease of the pouch, cuffitis, and irritable pouch syndrome [1, 2]. Pouchitis almost exclusively affects those with UC rather than FAP, and it usually responds well to treatment with antibiotics. However, pouchitis remains the most frequent complication of IPAA with a prevalence up to 50% and a significant impact on quality of life [1–3]. The majority of pouchitis cases are idiopathic but up to 20–30% of cases are found to have an identifiable cause including acute infections [4].

## 2. Case Presentation

**Figure 1:**
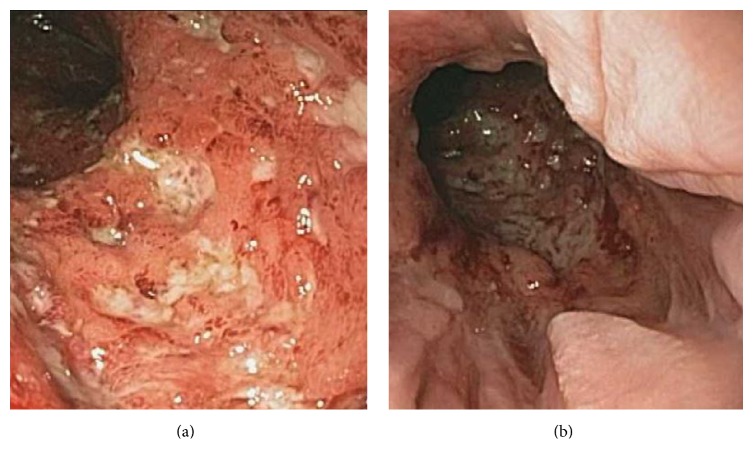
(a) Prepouch ileum. (b) Distal pouch.

A 41-year-old man with ulcerative colitis and a history of total proctocolectomy with subsequent IPAA for toxic megacolon in 1999 presented with fever, right lower quadrant abdominal pain, and diarrhea. Since the IPAA, he had mild recurrent acute pouchitis a few times per year, with symptoms of increased stool frequency and urgency, which would promptly resolve with 2-3 days of ciprofloxacin and metronidazole. His most recent episode of pouchitis was 4 months prior to presentation. Five days prior to presentation, he developed a fever up to 103°F and watery diarrhea every 20–30 minutes. Four days prior to presentation, he developed severe right lower quadrant abdominal pain that became progressively worse. Complete blood count, serum electrolytes, renal function, and liver function tests were normal except for mild acute kidney injury with creatinine 1.37 mg/dL. C-reactive protein was markedly elevated at 27 mg/dL. CT of the abdomen and pelvis with IV and PO contrast showed wall thickening of the distal ileum and pouch with no abscess. Due to his high fever, he was started on piperacillin-tazobactam empirically.* Clostridium difficile* PCR, ova and parasites, giardia stool antigen, and cryptosporidium stool antigen were all negative. He subsequently underwent pouchoscopy, which demonstrated an endoscopic pattern resembling Crohn's disease in the pouch and distal ileum with severe inflammation, marked friability, and deep ulcerations within the pouch and prepouch ileum (Figures 1(a) and 1(b)). However, histologically the small bowel mucosa was acutely inflamed with no evidence of chronic inflammation, architectural distortion, or granulomas. Stains for CMV and HSV were negative.

**Figure 2:**
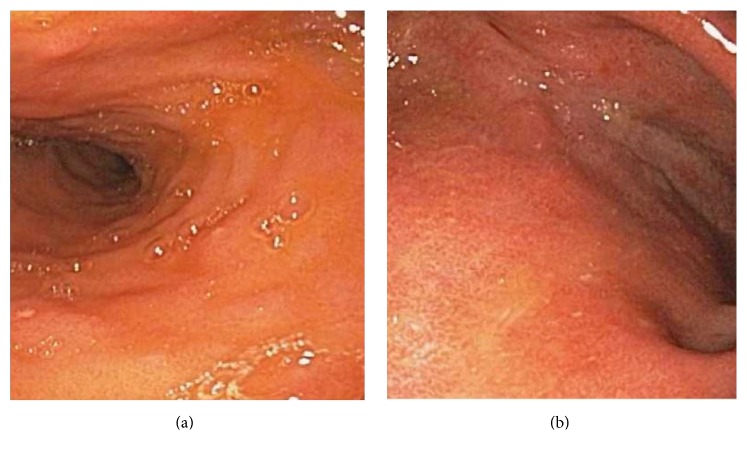
(a) Prepouch ileum. (b) Distal pouch.

On hospital day 4, a stool culture was positive for campylobacter and subsequently further characterized as* Campylobacter coli* by matrix assisted laser desorption ionization-time of flight (MALDI-TOF) mass spectrometry. At this point, the patient's stool frequency had improved to 7 bowel movements per day and he had defervesced on piperacillin-tazobactam. He was discharged on Azithromycin 500 mg by mouth daily for a total of 10 days to treat severe* Campylobacter coli* pouchitis. On follow-up 4 months later a pouchoscopy showed normal mucosa in the pouch and prepouch ileum (Figures 2(a) and 2(b)) and he continues to do well today.

## 3. Discussion

Campylobacter is an uncommon cause of pouchitis [5], but this case serves as a reminder to check for its presence in severe acute pouchitis. Patients with enteric infections from* Campylobacter* species typically present with symptoms of fever followed by abdominal pain, dehydration, and diarrhea and occasionally display endoscopic features that mimic Crohn's disease. Cases of campylobacter enterocolitis causing transmural inflammation resulting in small intestinal obstruction and toxic megacolon in patients without inflammatory bowel disease have been described [6, 7]. Two cases have been reported to date describing ulcerative colitis patients with ileal pouches affected by campylobacter-associated infection, both presenting with high fever, dehydration, abdominal pain, and increased bowel frequency [5]. In this case, the lack of histological evidence of chronic inflammation to suggest Crohn's disease would have raised suspicion for acute infectious ileitis even in the absence of a stool culture positive for campylobacter.
